# Region-specific susceptibility change in cognitively impaired patients with diabetes mellitus

**DOI:** 10.1371/journal.pone.0205797

**Published:** 2018-10-11

**Authors:** Mina Park, Won-Jin Moon, Yeonsil Moon, Jin Woo Choi, Seol-Heui Han, Yi Wang

**Affiliations:** 1 Department of Radiology, Konkuk University Medical Center, Konkuk University School of Medicine, Seoul, Republic of Korea; 2 Department of Neurology, Konkuk University Medical Center, Konkuk University School of Medicine, Seoul, Republic of Korea; 3 Department of Radiology, Weill Cornell Medical College, New York, New York, United States of America; UCLA, UNITED STATES

## Abstract

Emerging evidence suggests that diabetes mellitus (DM) is associated with iron and calcium metabolism. However, few studies have investigated the presence of DM in cognitively impaired patients and its effect on brain iron and calcium accumulation. Therefore, we assessed the effects of DM on cognitively impaired patients using quantitative susceptibility mapping (QSM). From June 2012 to Feb 2014, 92 eligible cognitively impaired patients underwent 3T magnetic resonance imaging (MRI). There were 46 patients with DM (DM+) and 46 aged matched patients without DM (DM-). QSM was obtained from gradient echo data and analyzed by drawing regions of interest around relevant anatomical structures. Clinical factors and vascular pathology were also evaluated. Measurement differences between DM+ and DM- patients were assessed by *t* tests. A multiple regression analysis was performed to identify independent predictors of magnetic susceptibility. DM+ patients showed lower susceptibility values, indicative of lower brain iron content, than DM- patients, which was significant in the hippocampus (4.80 ± 8.31 ppb versus 0.22 ± 10.60 ppb, p = 0.024) and pulvinar of the thalamus (36.30 ± 19.88 ppb versus 45.90 ± 20.02 ppb, p = 0.023). On multiple regression analysis, microbleed number was a predictor of susceptibility change in the hippocampus (F = 4.291, beta = 0.236, p = 0.042) and DM was a predictor of susceptibility change in the pulvinar of the thalamus (F = 4.900, beta = - 0.251, p = 0.030). In cognitively impaired patients, presence of DM was associated with lower susceptibility change in the pulvinar of the thalamus and hippocampus. This suggests that there may be region-specific alterations of calcium deposition in cognitively impaired subjects with DM.

## Introduction

With the continued aging of the world’s population, cognitive impairment has become a major medical and social problem. Although the most common cause of cognitive impairment in the elderly is Alzheimer’s disease (AD), its complex pathophysiology is not fully understood. Recently, vascular risk factors were suggested to be strongly associated with AD as well as vascular dementia [1]. Diabetes mellitus (DM), a known risk factor for cardiovascular disease, has been shown to have a strong link with cognitive impairment associated with AD [2,3]. However, the exact pathomechanism of how DM influences AD progression is still unknown.

Increasing evidence from magnetic resonance imaging (MRI) studies suggests that there is brain iron overload in various neurodegenerative diseases [4], such as AD [5,6] and multiple sclerosis (MS) [7]. Likewise, there is also a considerable amount of evidence suggesting that calcium deposition increases with age and in the many neurodegenerative diseases that show calcium dysregulation, such as parathyroid disorders, Fabry disease, Fahr disease, and AD [8–11].

DM is thought to be related to dysregulation and accumulation of either iron, calcium, or both. In an experimental study, rats with diabetes were found to have increased calcifications in the thalamus and basal ganglia compared to normal controls [12]. Even in human subjects, DM is suggested to be related to calcium deposition in deep gray matter [13]. At the same time, DM is known to be associated with systemic iron overload [14], although the relationship between DM and brain iron concentration is not straightforward due the presence of the blood-brain barrier [4,15]. Given the close association between DM and cognitive impairment [2,3], the presence of DM may exacerbate the accumulation of iron or calcium in the brains of cognitively impaired patients, which can be visualized using appropriate *in vivo* imaging methods.

Recently, quantitative susceptibility mapping (QSM) has been suggested as an imaging tool to evaluate iron and/or calcium in tissues [16,17]. While iron shows positive susceptibility, calcium deposition shows negative susceptibility due to its diamagnetic property [18]. As QSM is less influenced by the background field (unlike traditional quasi-gold standard T2* mapping [17,19,20]), it is being increasingly used to assess brain iron [5,17,19,21] and calcium levels *in* vivo [16].

We hypothesized that either iron or calcium deposition that can be measured by QSM would be altered differentially in the brains of cognitively impaired patients with DM compared to those without DM. If calcium deposition is the predominant pathology due to DM, QSM would reveal more negative susceptibility. If iron deposition is the predominant pathology due to DM, QSM would reveal more positive susceptibility compared with non-DM subjects. Therefore, our goal was to measure brain susceptibility in patients with cognitive impairment and to evaluate the effect of DM on clinical factors and vascular pathology.

## Materials and methods

This retrospective analysis of prospectively acquired data was reviewed and approved by our institutional review board of Konkuk University Medical Center (#KUH1140040). All clinical investigations were conducted according to the principles expressed in the Declaration of Helsinki. The requirement to obtain informed consent was waived by our institutional review board because the data were analyzed anonymously.

### Subjects

There were 229 consecutive patients who were referred from a community-based dementia center and underwent brain MRIs at 3T from June 2012 to Feb 2014 for subjective memory complaints. Among them, 83 subjects with other forms of dementia, Parkinson’s disease, or other neurologic disease, 9 with unavailable raw data, and 23 with incomplete study were excluded. From 114 final eligible subjects who were diagnosed with cognitive impairment due to AD or mild cognitive impairment (MCI), 46 cognitively impaired subjects with DM and 46 age-matched cognitively impaired subjects without DM were included in this study. We assessed basic demographic characteristics, other medical conditions (including vascular risk factors), laboratory test results, global cognitive assessments (Clinical Dementia Rating Scale sum of boxes and Mini-Mental Status Examination scores, and Geriatric Depression Scale [GDepS] values), and brain MRI scans. The results of laboratory tests were used to exclude other medical conditions associated with dementia-like symptoms [5]. The vascular risk factors were selected based on a previous study [22]. All data were collected between the first and second visit (within 1–2 months). Diagnoses of dementia, probable AD, and mild cognitive impairment (MCI) were based on the Diagnosis and Statistical Manual of Mental Disorders (4^th^ ed.), the criteria of the National Institute of Neurological and Communicative Disorders and Stroke and the Alzheimer’s Disease and Related Disorders Association (NINCDS-ADRDA) [23], and the criteria suggested by Peterson et al. [24].

### MRI acquisition

All patients underwent MRI using a 3T unit (Signa HDxT; GE Healthcare, Milwaukee, WI) with an 8-channel head coil. The routine MRI protocol included the following sequences: 1) axial and sagittal T1-weighted inversion-recovery (repetition time [TR]/echo time [TE]/ inversion time [TI], 2468/12/920 ms; section thickness, 5 mm; matrix, 512 × 224); 2) axial T2-weighted fast spin-echo (FSE) (TR/effective TE, 4000/106 ms; section thickness, 5 mm; matrix, 384 × 384); 3) axial fluid-attenuated inversion-recovery (TR/TE/TI, 11,000/105/2600 ms; sectional thickness, 5 mm; matrix 384 × 224); 4) axial T2-weighted gradient-echo (GRE) (TR/TE, 550/17 ms; section thickness, 5 mm; matrix, 384 × 224; flip angle, 15°); and 5) T1-weighted volumetric fast spoiled gradient recalled-echo (FSPGR) (TR/TE, 7.3/2.7 ms; section thickness, 1.5 mm; matrix, 256 × 256; flip angle, 13°). The field of view (FOV) was 230 × 230 mm.

For T2*-weighted imaging, we acquired a multi-echo axial three-dimensional gradient echo sequence (based on susceptibility-weighted angiography sequences) with the following parameters: TR/TE = 37/3.5 ms, 8 echoes with echo spacing = 4.09 ms, FA = 20°, bandwidth = ±41.67 kHz, FOV = 240 × 240 mm^2^, matrix = 256 × 256, in-plane resolution = 0.938 × 0.938 mm^2^, slice number = 56, slice thickness = 2.5 mm, and acquisition time = 3 min 32 s.

### Quantitative analysis for QSM

The QSM images were pre-processed and analyzed using a tool box and a standardized algorithm as suggested by Liu T et al [25]. QSM images were reconstructed by using the morphology enabled dipole inversion (MEDI) approach, which inverts an estimated magnetic field to generate a magnetic susceptibility distribution that is structurally consistent with an anatomic prior derived from the magnitude image obtained during the same imaging [26]. First, a nonlinear fitting for the multi-echo data was performed to estimate the magnetic field inhomogeneity, followed by a magnitude-guided phase unwrapping [25]. The background field was then removed by applying the projection onto the dipole field (PDF). Finally, the remaining file was inverted to calculate QSM by using MEDI [27]. QSM values were referenced to cerebrospinal fluid of the lateral ventricular atrium to allow comparison across subjects [28]. For accurate region of interest (ROI) placement, 3D T1-weighted images were co-registered to the corresponding QSM images of each subject by using a normalized mutual information-based method [29] with the software tool, Statistical Parametric Mapping (SPM) version 8 (SPM8, http://www.fil.ion.ucl.ac.uk/spm/).

**Fig 1:**
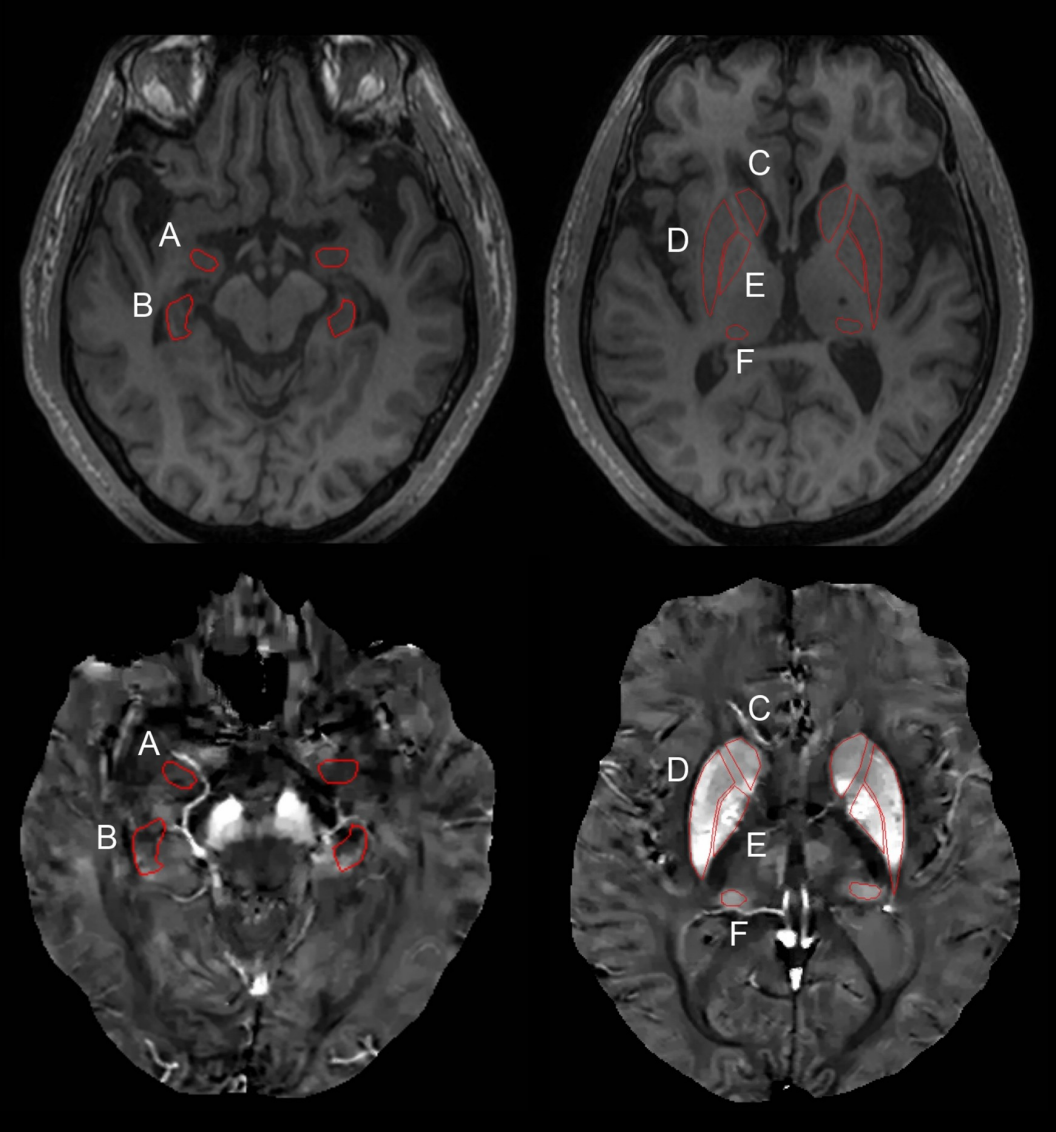
**ROI placement of QSM images: A. amygdala; B. hippocampus; C. caudate nucleus; D. putamen; E. globus pallidus; F. thalamus (pulvinar nucleus).** For inter-observer reliability, another rater (rater B) independently measured the susceptibility value of semiautomatically drawn ROIs. Inter-rater reliability among all regions was 0.960 (95% confidence interval [CI]: 0.944–0.971, p < 0.001).

ROI placement was performed using MIPAV version 7.2.0 (Medical Imaging Processing, Analysis, and Visualization, http://mipav.cit.nih.gov). Six ROIs were drawn semi-automatically on the 3D-T1-weighted images and then were transferred to the co-registered QSM images for analysis. The inclusion of vessels was avoided when the ROIs were drawn. The six ROIs were: the amygdala, hippocampus, caudate nucleus, globus pallidus, putamen, and pulvinar nucleus of the thalamus (Fig 1). ROI placement was done on the axial reconstructed images at the level of 12 mm above the anterior commissure-posterior commissure line. For the hippocampus (body) and amygdala, the axial slices of co-registered T1-weighted images that showed the mammillary body best were selected [30]. Since the boundary of the thalamic pulvinar nucleus was not identifiable on MRI alone, we operationally defined the thalamic pulvinar nucleus using a level-set ROI tool of MIPAV. Additionally, an ROI for the lateral ventricular atrium for the reference of QSM values was also placed. The average susceptibility value of each ROI after subtracting the value of the lateral ventricular atrium from the average value of each ROI was used for subsequent statistical analyses. An experienced rater (rater A) with 3 years of experience in neuroimaging processing performed the entire imaging analysis under the supervision of an experienced neuroradiologist (W.M.). The quality of co-registration and ROI placement was checked by one neuroradiologist on the basis of anatomical landmarks.

### Imaging analysis for vascular risk factor

An experienced neuroradiologist assessed the vascular risk factor on MRI. White matter hyperintensity (WMH) was defined as lesions of white matter hyperintensity on fluid-attenuated inversion recovery (FLAIR) images according to the STandards for ReportIng Vascular changes in nEuroimaging (STRIVE) criteria [31], and graded according to the Fazekas scale as deep WMH (0 = absent; 1 = punctate; 2 = early confluent; and 3 = confluent) and periventricular WMH (0 = absent; 1 = caps or pencil-thin lining; 2 = smooth halo; 3 = irregular WMH extending into deep white matter) [32]. A total Fazekas score was calculated by adding the periventricular and deep WMH scores; when it was greater than 3, the status was designated as WMH positive. To assess the inter-observer reliability of the Fazekas scale, another rater independently determined the Fazekas score of 20 subjects from our study data, and the resultant intraclass correlation coefficient was 0.911 (95% CI, 0.855–0.946, p < 0.001). Lacunes were defined as small lesions (3–15 mm in diameter) that were hypointense on T1-weighted images, hyperintense on T2-weighted images, and had a perilesional halo on FLAIR images [31]. Microbleeds (MBs) were defined as small signal voids (≤10 mm in diameter) with associated blooming on T2*-weighted images [31]. Both the number and presence of WHM, lacunes, and MBs were recorded according to previously described definitions.

### Statistical analysis

Statistical analysis was performed using the Statistical Package for the Social Sciences (Version 21.0 for Windows; SPSS, Chicago, Ill) and MedCalc (Version 15.2.2, Mariakerke, Belgium). A p value of <0.05 was considered to be statistically significant. Before performing individual analyses, the distributions of data sets were checked for normality.

To compare the clinical and radiologic features including magnetic susceptibility within a specific ROI according to the presence of DM, we used the unpaired t-test or Mann–Whitney U test for continuous variables and the chi-square test or Fisher’s exact test for categorical variables.

Pearson and Spearman correlation tests were performed to explore the relationships between clinical demographic variables and vascular factors on susceptibility values of regions with significant differences.

To evaluate the combined effect of one or more explanatory variables on the susceptibility of the hippocampus and pulvinar nucleus of the thalamus, which were the only two regions with significant susceptibility differences in accordance to the presence of DM, we performed a multiple linear regression analysis with a forward stepwise approach. Collinearity was controlled by eliminating the independent variables showing multi-collinearity. In the multiple regression analysis, presence of DM and significant variables of the group comparison and aforementioned correlation tests were included as independent variables.

To assess inter-rater reliability, agreement between the two raters was determined using the intraclass correlation coefficient.

## Results

Among the included 92 cognitively impaired patients, 46 patients had DM and 46 had no DM. In DM+ group, 40 (87.0%) were AD patients and in the DM- group, 34 (73.9%) were AD patients (p = 0.115).

In terms of clinical risk factors (Table 1), hypertension and hyperlipidemia were more frequently found in DM+ patients (p = 0.003), and DM+ patients had significantly higher GDepS scores (p = 0.023).

**Table 1:** Clinical and demographic data comparisons between cognitively impaired patients with or without diabetes mellitus.

	No DM (n = 46)	DM (n = 46)	P value
Sex (female)	41 (89.1%)	36 (78.3%)	0.158
Hypertension	18 (39.1%)	32 (69.6%)	0.003*
Hyperlipidemia	11 (23.9%)	19 (41.3%)	0.075
CVA History	4 (8.7%)	5 (10.9%)	1.000
Age, years	77.6 ± 8.7	76.2 ± 8.1	0.424
Education, years	4.5 ± 4.9	5.2 ± 5.2	0.543
MMSE	15.9 ± 6.3	18.0 ± 6.0	0.113
CDR SOB*	5.28 ± 4.27	3.78 ± 2.72	0.292
GDepS*	5.60 ± 4.54	8.03 ± 4.92	0.023*

CDR: Clinical Dementia Rating. CDR SOB: Clinical Dementia Rating–sum of boxes. CVA: cerebrovascular accident. DM: diabetes mellitus. GDepS: General Depression Scale. MMSE: Mini Mental Status Examination

* indicates P < 0.05

The vascular factors detected by MRI (Table 2), such as the presence of WMH, lacunes, and MBs did not differ between DM+ and DM- patients.

**Table 2:** Comparisons of vascular factors detected by MRI between cognitively impaired patients with or without diabetes mellitus.

	No DM (n = 46)	DM (n = 46)	P value
Fazekas DW score	1.5 ± 0.9	1.3 ± 0.9	0.385
Fazekas PW score	1.2 ± 0.9	1.3 ± 1.0	0.485
Total Fazekas score	2.7 ± 1.7	2.7 ± 1.7	0.924
Lacune No.	0.2 ± 0.8	0.2 ± 0.9	1.000
MB No.	2.0 ± 5.7	0.9 ± 3.3	0.702
Presence of WMH	22 (47.8%)	25 (54.3%)	0.532
Presence of lacunes	5 (10.9%)	5 (10.9%)	1.000
Presence of MBs	8 (17.4%)	7 (15.2%)	0.778

DM: diabetes mellitus. DW: deep white matter. MB: microbleed. PW: periventricular white matter. WMH: white matter hyperintensity

**Fig 2:**
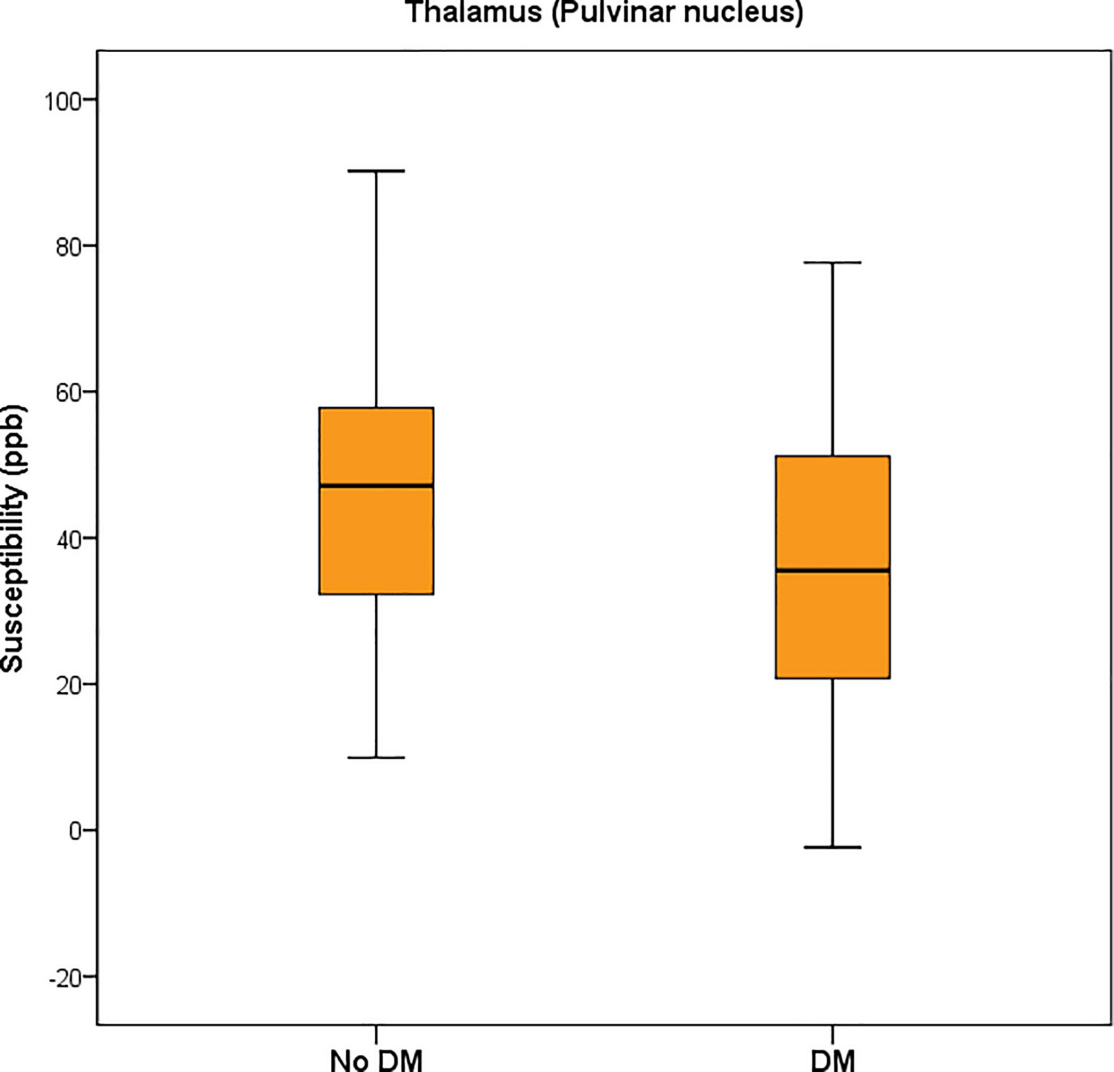
Magnetic susceptibility in the hippocampus of patients with and without DM.

**Fig 3:**
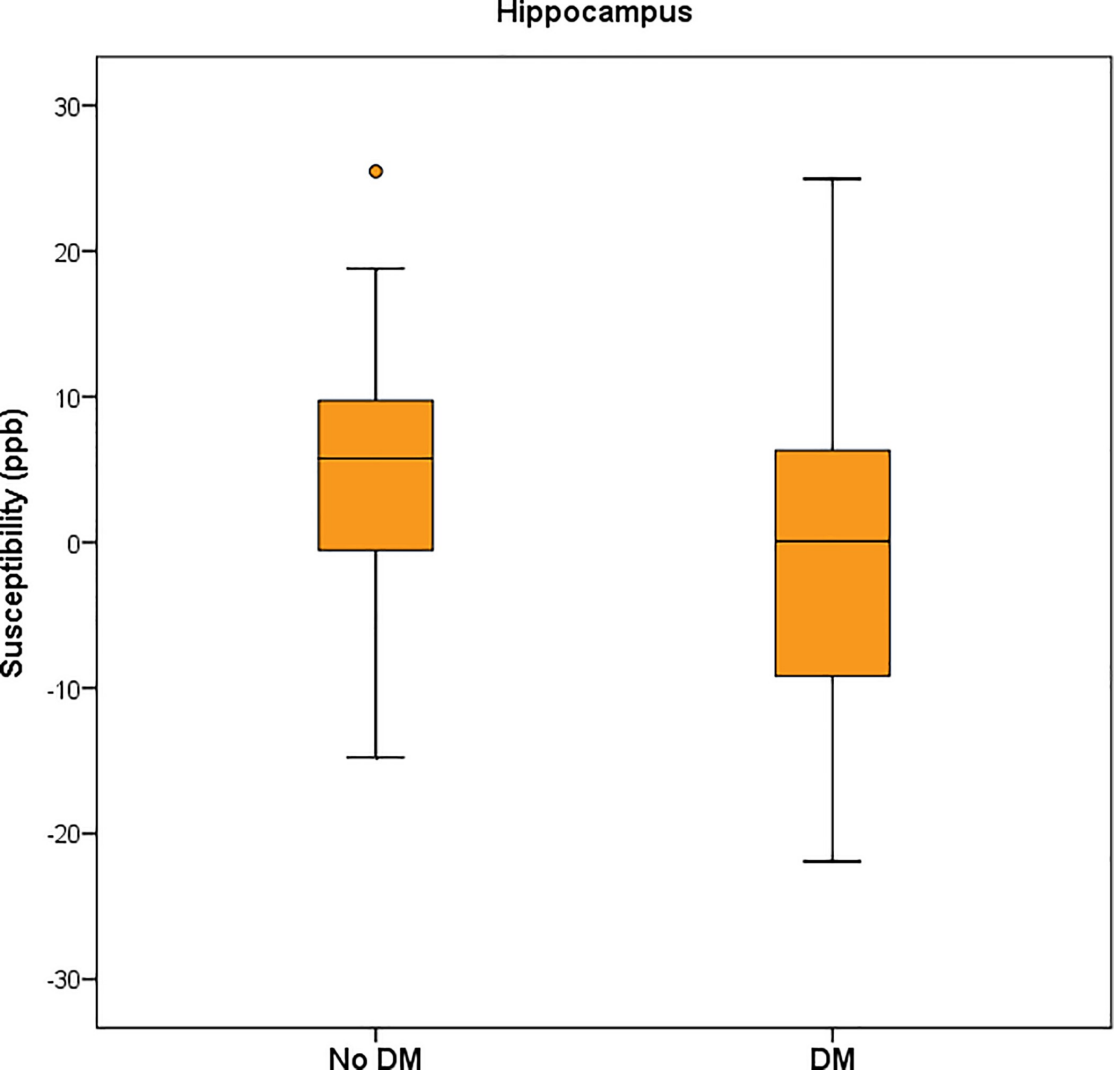
Magnetic susceptibility in the thalamus of patients with and without DM.

**Fig 4:**
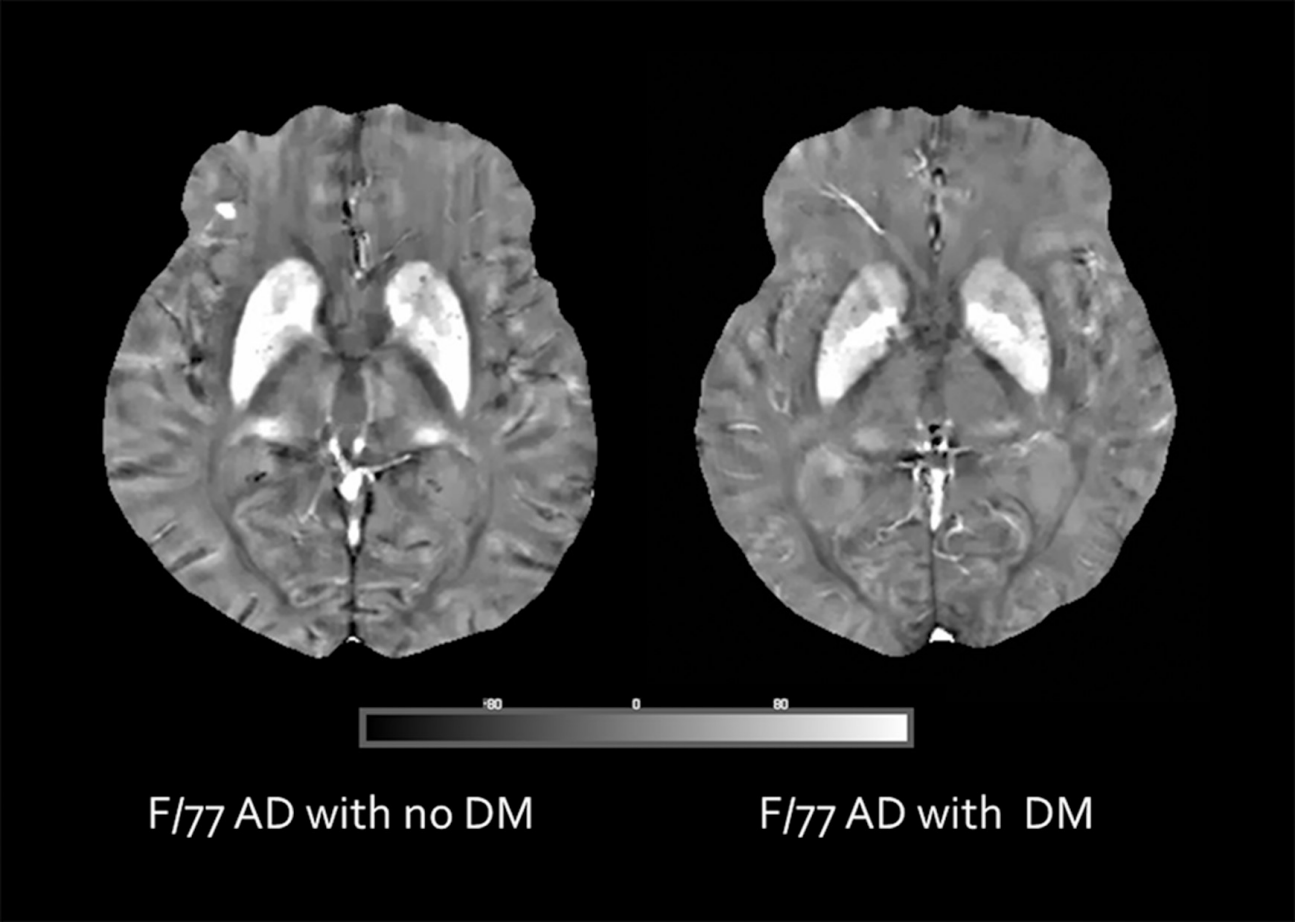
Brain magnetic susceptibility difference in DM-positive and DM-negative cognitively impaired patients. Susceptibility values are in SI units (parts per billion, ppb).

The iron content of different areas of the brain was indicated by the magnetic susceptibility value measured by the QSM technique. The hippocampus showed significant lower susceptibility in the DM+ group compared to the DM- group (4.80 ± 8.31 ppb versus 0.22 ± 10.60 ppb, p = 0.024) (Fig 2). The left hippocampus also showed significant lower susceptibility in the DM+ compared to the DM- group (p = 0.015). Susceptibilities of the thalami were lower in the DM+ group as compared to those in the DM- group (p = 0.023) (Fig 3). The mean susceptibility in the right and left pulvinar nuclei of the thalamus was significantly lower in the DM+ group than in that the DM- group (47.09 ± 22.85 ppb versus 37.30 ± 20.23 ppb, p = 0.032 and 44.70 ± 20.06 ppb versus 33.30 ± 21.19 ppb, p = 0.032, respectively) (Fig 4). There was no significant difference of susceptibility in the other brain regions between the two groups (Table 3).

**Table 3:** Comparison of susceptibility values in signal intensity units (parts per billion) of each anatomical regions of brain between cognitively impaired patients with or without diabetes mellitus.

Susceptibility (ppb)	No DM (n = 46)	DM (n = 46)	P value
Amygdala	-9.10 ± 11.16	-5.75 ± 14.05	0.679
Left amygdala	-10.73 ± 11.65	-6.84 ± 17.45	0.212
Right amygdala	-7.47 ± 14.67	-4.65 ± 16.76	0.393
Hippocampus	4.80 ± 8.31	0.22 ± 10.60	0.024*
Left hippocampus	5.64 ± 10.45	-0.78 ± 14.22	0.015*
Right hippocampus	3.95 ± 10.43	1.23 ± 11.40	0.235
Caudate	88.83 ± 26.84	96.75 ± 27.89	0.179
Left caudate	87.54 ± 29.18	99.78 ± 29.83	0.050
Right caudate	90.12 ± 26.64	93.73 ± 28.35	0.531
Globus pallidus	148.22 ± 55.64	148.94 ± 55.97	0.882
Left globus pallidus	146.22 ± 63.28	146.09 ± 52.64	0.992
Right globus pallidus	150.22 ± 49.96	151.79 ± 62.91	0.895
Putamen	114.64 ± 36.95	122.95 ± 44.02	0.382
Left putamen	113.30 ± 36.06	120.92 ± 46.22	0.381
Right putamen	115.98 ± 41.15	124.99 ± 43.71	0.311
Thalamus (pulvinar)	45.90 ± 20.02	36.30 ± 19.88	0.023*
Left thalamus (pulvinar)	44.70 ± 20.06	33.30 ± 21.19	0.032*
Right thalamus (pulvinar)	47.09 ± 22.85	37.30 ± 20.23	0.032*

DM: diabetes mellitus; ppb: parts per billion

* indicates P < 0.05

In the whole population, the mean susceptibility of the bilateral hippocampus and right hippocampus were positively correlated with the total Fazekas score (rho = 0.240, p = 0.021 and rho = 0.291, p = 0.005, respectively) and number of MBs (rho = 0.243, p = 0.020 and rho = 0.248, p = 0.017, respectively). The left hippocampus showed no significant correlation between its mean susceptibility and any clinical assessment scales or vascular factors detected by MRI.

The mean susceptibility of the bilateral pulvinar of the thalamus and left pulvinar nucleus of the thalamus did not correlate to any clinical assessment scales or vascular factors detected by MRI. However, the mean susceptibility of the right pulvinar nucleus of the thalamus tended to be negatively correlated with the scores in the geriatric depression scale (rho = -0.267, p = 0.020).

Multiple regression analysis revealed that DM was the only reliable predictor of susceptibility change in the bilateral pulvinar of the thalamus (F = 4.900, beta = - 0.251, p = 0.030) while number of microbleeds was the only significant predictor of susceptibility change in the bilateral hippocampus (F = 4.291, beta = 0.236, p = 0.042).

## Discussion

The comparison of imaging data between DM+ and DM- cognitively impaired patients demonstrated that the presence of DM did not affect cerebrovascular pathology, but it did cause a decrease in the magnetic susceptibility of the hippocampus and pulvinar of the thalamus. This suggests that brain iron and/or calcium contents are altered by the presence of DM in a region-specific manner in patients with cognitive impairment.

In light of the brain iron dysmetabolism in patients with DM, we initially hypothesized that, given the known systemic iron overloading effects of DM [14], brain iron content would be increased in the DM+ group. Conversely, we observed decreased susceptibility in the brain in the DM+ group.

Until now, previous studies investigating the iron content in the pulvinar nuclei of patients with neurodegenerative diseases have shown inconsistent results [5,20]. While the majority of these studies reported increased brain iron content in certain pathophysiological states, some studies have shown iron load decreasing during the chronic stage of MS, or in the deep gray matter of patients with severe ischemic white matter disease [7,33,34]. The latter findings are consistent with our observations of lower susceptibility in the presence of DM, which is a known trigger factor in neurodegeneration and ischemic white matter disease [1]. Moreover, recent publications confirmed a decreased susceptibility of the thalamus, including the pulvinar nuclei in MS patients, that further decreased over time [35,36], which is in line with our observations. Brain iron mostly exists as the iron storage protein ferritin, and is found in oligodendrocytes and neurons [4,37]. The decreased susceptibility we observed might therefore be due to chronic microglia activation causing a depletion of iron from oligodendrocytes in the thalamus, as inflammation-mediated iron release may lead to a vicious cycle that reduces the protection of axons and neuronal repair [36]. Furthermore, iron depletion combined with relatively increased myelin density from gray matter atrophy might also explain the decreased thalamic susceptibility [35].

However, our results contradict a recent *in vivo* human study which investigated the association between brain iron content and the preclinical stage of DM [15]. The previous study found that increased brain iron contents in the caudate, hypothalamus, and lenticular nucleus were negatively associated with cognitive performance in obese subjects at risk for impaired glucose metabolism. This discrepancy with our results suggests that there may be differences in the clinical status of subjects in terms of glucose dysmetabolism between the two studies or that there may be another factor affecting brain susceptibility instead of iron in this study.

Accordingly, we proposed a second hypothesis that calcium was responsible for the susceptibility change observed in this study. Considering the commonality of brain calcium accumulation in a variety of neurodegenerative diseases, the reduced susceptibility seen in DM subjects in this study may be due to increased calcium rather than iron depletion. Calcium accumulated in the brain takes the form of hydroxyapatite, which has a volume susceptibility of -14.83 ppm and is slightly diamagnetic relative to water [38]. Therefore, calcium accumulation can contribute to the decreased susceptibility seen in the QSM images. Basal ganglia and thalamic calcification or calcium accumulation is frequently observed in many neurodegenerative disorders: Down syndrome, hypoxia-ischemia, and physiologic aging [8–11]. In a study using diabetic rats, basal ganglia and thalamic calcifications were noted even without microscopically significant vascular or parenchymal abnormalities, which is in line with our observation of decreased susceptibly in DM patients without cerebrovascular pathology [12]. Hippocampal calcification is also a common finding in older adults and especially in the patients with cognitive problems, in association with risk factors of vascular disease [39–41]. It may partially explain our observation of decreased susceptibility in the hippocampus of DM+ cognitively impaired patients and its significant correlations with the total Fazekas score and MBs, which represent imaging vascular risk factors [31]. According to a recent experimental study on post-status epilepticus neurodegeneration, QSM can visualize the deposition of both iron and calcium in bilateral thalamic regions, a finding correlated with histopathologic results [16]. Although it remains uncertain how brain susceptibility will change when both iron and calcium co-exist within the same voxel, we suggest that calcium deposition–rather than iron depletion–may be more responsible for susceptibility change in deep gray matter, based on the evidence provided by this study.

Regarding other brain regions beside the thalamus and hippocampus, it is unclear why there was no difference in basal ganglia susceptibility between DM and non-DM subjects. We can only speculate that differential geographic involvement of calcium deposition may be the underlying cause of this finding. Another possibility is that calcium depositions occur concomitantly with iron deposition and their differential deposition may have cancelled each other’s susceptibility change. In DM subjects, prominent iron accumulation in the basal ganglia might have been masked due to more prominent calcium deposition, which could lead to no differences, as compared with the findings in non-DM subjects.

Regarding the relationship between susceptibility and clinical factors, we found that there was a significant correlation between susceptibility of the right pulvinar nucleus and GDepS values in the DM+ group. Higher GDepS values (indicating severe depression) were associated with lower susceptibility in the DM+ group. There is a substantial amount of evidence that the thalamus—especially the pulvinar nucleus—is associated with major depression disorder (MDD) [42]. Thus, we suggest that lower susceptibility in the DM+ group may be used as an imaging marker for predicting the presence of depression in cognitively impaired subjects.

Interestingly, we did not find any difference in WMH, lacunes, or MBs between the DM+ or DM- groups. Our findings support those of a previous study showing no difference in small vessel ischemic disease between DM+ and DM- groups [43]. Conversely, our result contradicts the finding of a large autopsy study that found an association between DM and a higher risk of brain infarcts, and in particular, lacunes [44]. However, unlike the previous study, our study was based on DM+ cognitively impaired patients, so a difference in the study population may have caused a variation in the results. Because it is still unclear whether DM affects the cerebrovascular pathologies differently according to cognitive status, further study is needed.

Multiple linear regression analysis revealed that only DM is associated with a change in thalamic susceptibility after controlling for all relevant clinical and imaging variables. We speculate that DM modulates thalamic susceptibility, potentially through increased calcium accumulation or through decreased iron accumulation [12,45].

There are several limitations to this study. First, we retrospectively evaluated the clinical status of patients from a community-based dementia center through questionnaires and the referring physicians’ diagnoses. Thus, we could not evaluate clinically important variables such as DM duration, DM medication, glycated hemoglobin (Hb1C), or especially, the *APOE4* allele, which is a risk-factor gene for AD. Therefore, a large-scale prospective study is needed to confirm the regional magnetic susceptibility changes and further explore the underlying mechanisms and the role of DM pathology in cognitive decline. Second, we performed semiautomatic manual segmentation for ROI placement. Although automatic segmentation is regarded as the best way for imaging analysis, we believe that manual segmentation can be the reference standard for complex structures such as the pulvinar nucleus [46].

In conclusion, the presence of DM is not associated with cerebrovascular pathology or brain volume, but may be associated with lower susceptibility (more calcium accumulation) in the left pulvinar nucleus of the thalamus as measured by QSM. Our results indicate that there may be region-specific alterations of calcium accumulation in cognitively impaired patients with DM.

## Supporting information

S1 Dataset (SAV)
